# Supplementary material for: Sigmoni: classification of nanopore signal with a compressed pangenome index
Source: Bioinformatics. 2024 Jun 28;40(Suppl 1):i287–96. doi: 10.1093/bioinformatics/btae213 (PMC11211819; doi:10.1093/bioinformatics/btae213)
Supplement: btae213_Supplementary_Data [file btae213_supplementary_data.zip › btae213_Supplementary_Data/Shivakumar.206.sup.1.pdf]

Sigmoni: classification of nanopore signal with a compressed pangenome index

Vikram S. Shivakumar[1,*], Omar Y. Ahmed[1], Sam Kovaka[1], Mohsen Zakeri[1], and Ben Langmead[1,*]

[1]Department of Computer Science, Johns Hopkins University
[*]*corresponding authors:* vshivak1@jhu.edu, langmea@cs.jhu.edu

Supplementary Tables

Table S1: Comparison of full-read mapping speed and accuracy between signal-based methods and the standard basecalled approach. 10,000 reads were simulated using Squigulator [1] from the Zymo mock community. The best performer across the signal-based tools in each metric is bolded. The signal-based methods were each run on a single thread, and the Guppy-CPU method was run with 48 threads. All analyses were performed on a 3 GHz Intel Xeon Gold Cascade Lake 6248R CPU.

	Precision	Recall	F1	Time (s)	Threads
Sigmoni	0.999	**0.997**	**0.998**	**146.7**	1
Sigmap	**1.0**	0.944	0.971	910.3	1
UNCALLED	0.999	0.992	0.996	174.6	1
RawHash2	**1.0**	0.978	0.989	205.8	1
Guppy + minimap2 (cpu)	1.0	1.0	1.0	9301.1	48

Supplementary Figures

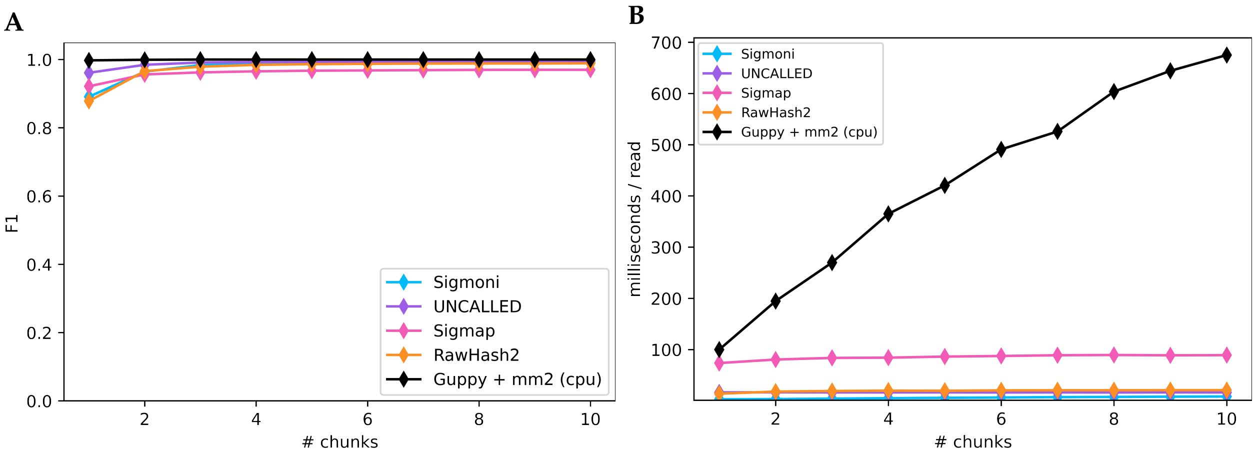

Figure S1: Comparison of mapping accuracy **(A)** and speed **(B)** on simulated reads from the Zymo mock community. The signal-based methods were run on a single thread, and Guppy-CPU was run with 48 threads. All analyses were run on a 3 GHz Intel Xeon Gold Cascade Lake 6248R CPU.

References

1. Gamaarachchi, H., Ferguson, J. M., Samarakoon, H., Liyanage, K. & Deveson, I. W. Squigulator: simulation of nanopore sequencing signal data with tunable noise parameters. *bioRxiv*, 2023–05 (2023).
